# Supplementary material for: Catalyst Design: Counter Anion Effect on Ni Nanocatalysts Anchored on Hollow Carbon Spheres
Source: Nanomaterials (Basel). 2023 Jan 20;13(3):426. doi: 10.3390/nano13030426 (PMC9919602; doi:10.3390/nano13030426)
Supplement: Supplementary file 1 [file nanomaterials-13-00426-s001.zip › nanomaterials-2166281-supplementary.pdf]

Supplementary Information

Catalyst design: Counter anion effect on Ni nanocatalysts anchored on hollow carbon spheres

Ryan O'Connor[1, 2], Joyce B. Matsoso[2, 3#], Victor Mashindi[2], Pumza Mente[2, 4§], Lebohang Macheli[5], Beatriz D. Moreno[6], Bryan P. Doyle[5], Neil J. Coville[1, 2*], Dean Barrett[1,2*]

[1]DSI-NRF Centre of Excellence in Strong Materials and [2]Molecular Science Institute, School of Chemistry, University of the Witwatersrand, WITS 2050, Johannesburg, South Africa

[3] Department of Inorganic Chemistry, University of Chemistry and Technology in Prague, Dejvice 6, 166 28 Prague 6, Czech Republic#

[4]Institute of Physical Chemistry, Polish Academy of Science, Warsaw, Poland§

[5]Department of Physics, University of Johannesburg, P.O. Box 524, Auckland Park 2006, South Africa

[6]Canadian Light Source Inc., 44 Innovation Boulevard, Saskatoon, SK S7N 2V3, Canada

Results

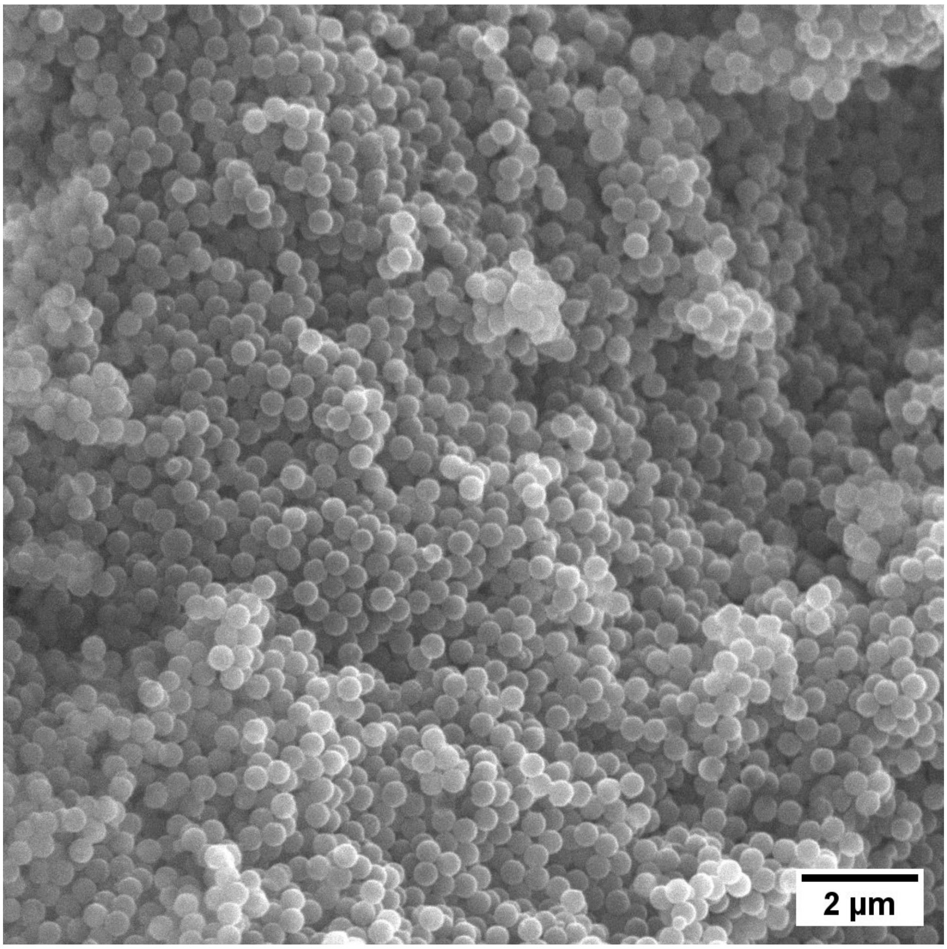

Figure S1: SEM micrographs of the PSS template.

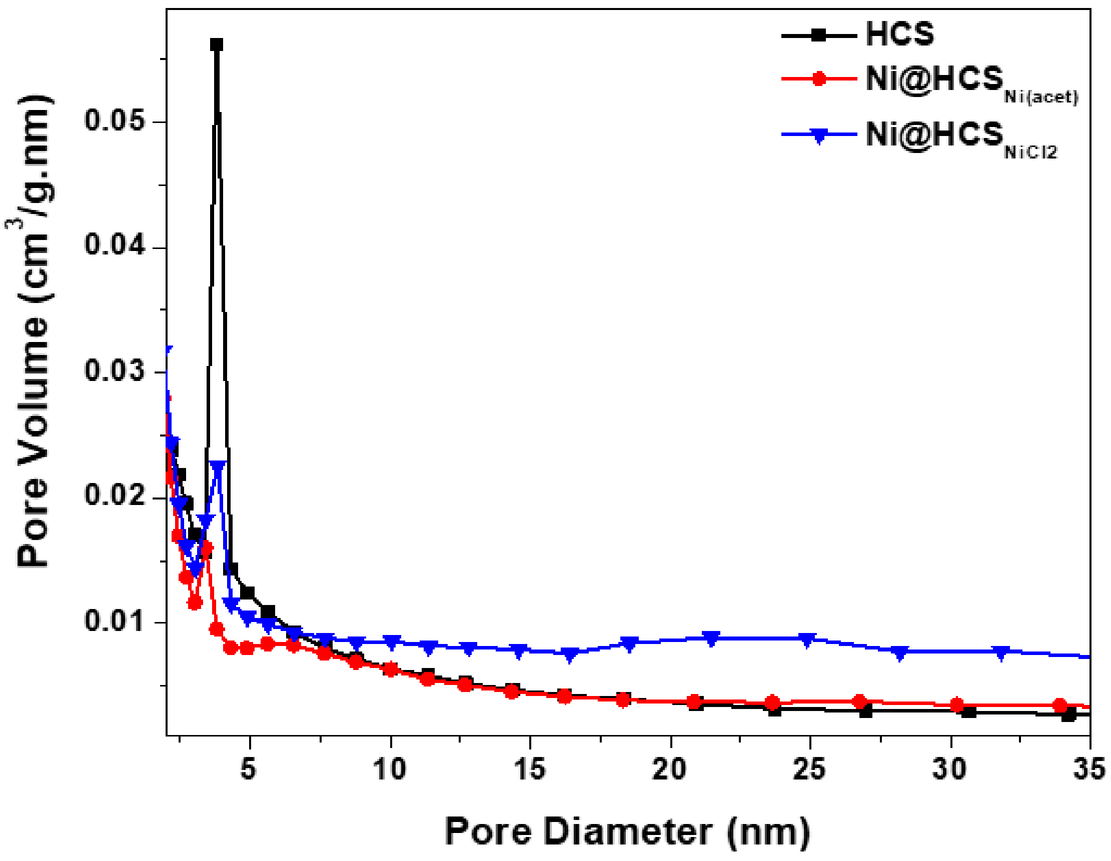

Figure S2: Pore size distributions of the pristine HCSs and Ni@HCSs nanocatalysts.

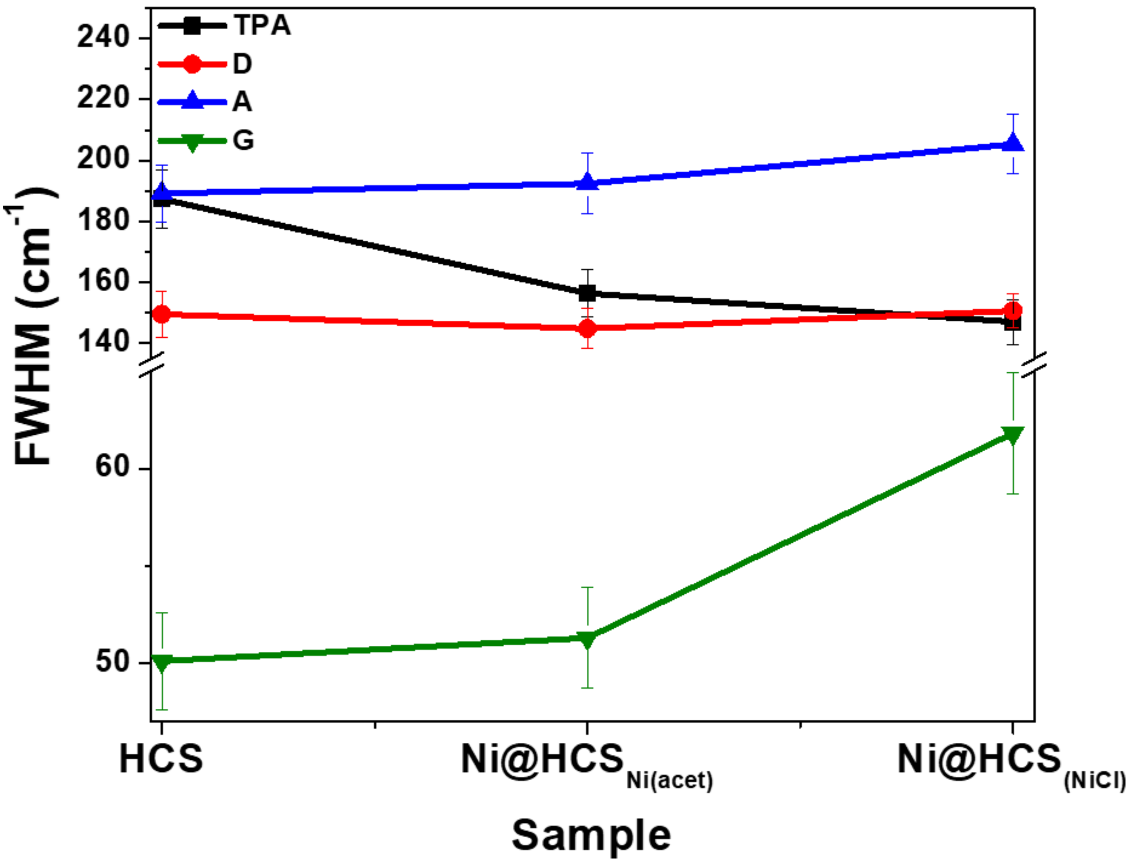

Figure S3: Peak broadening for all Raman-active bands for the pristine HCSs and nanocatalysts.

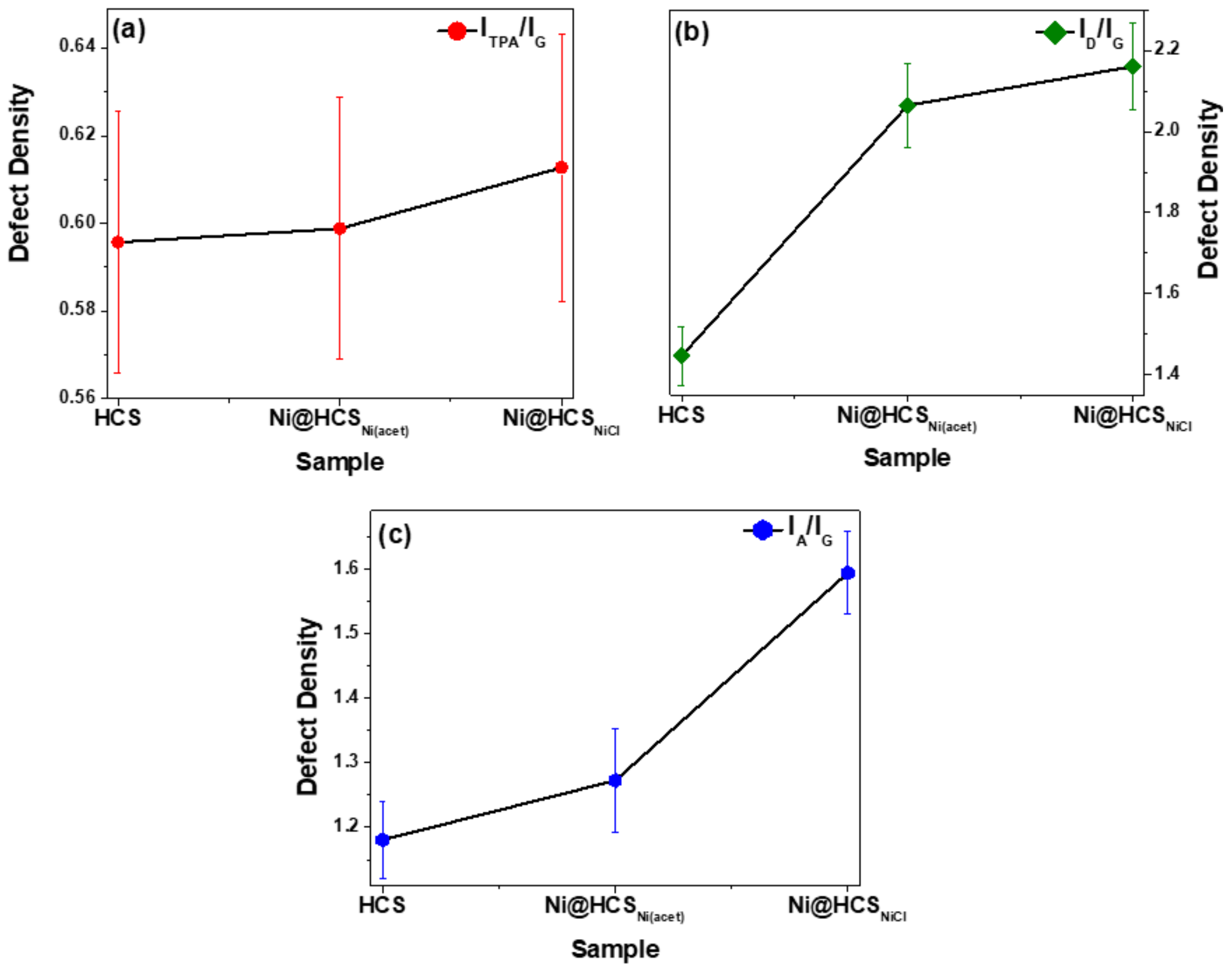

Figure S4: Defect density ratios (a) I_{TPA}/I_G, (b) I_D/I_G and (c) I_A/I_G of the pristine and Ni@HCSs samples.

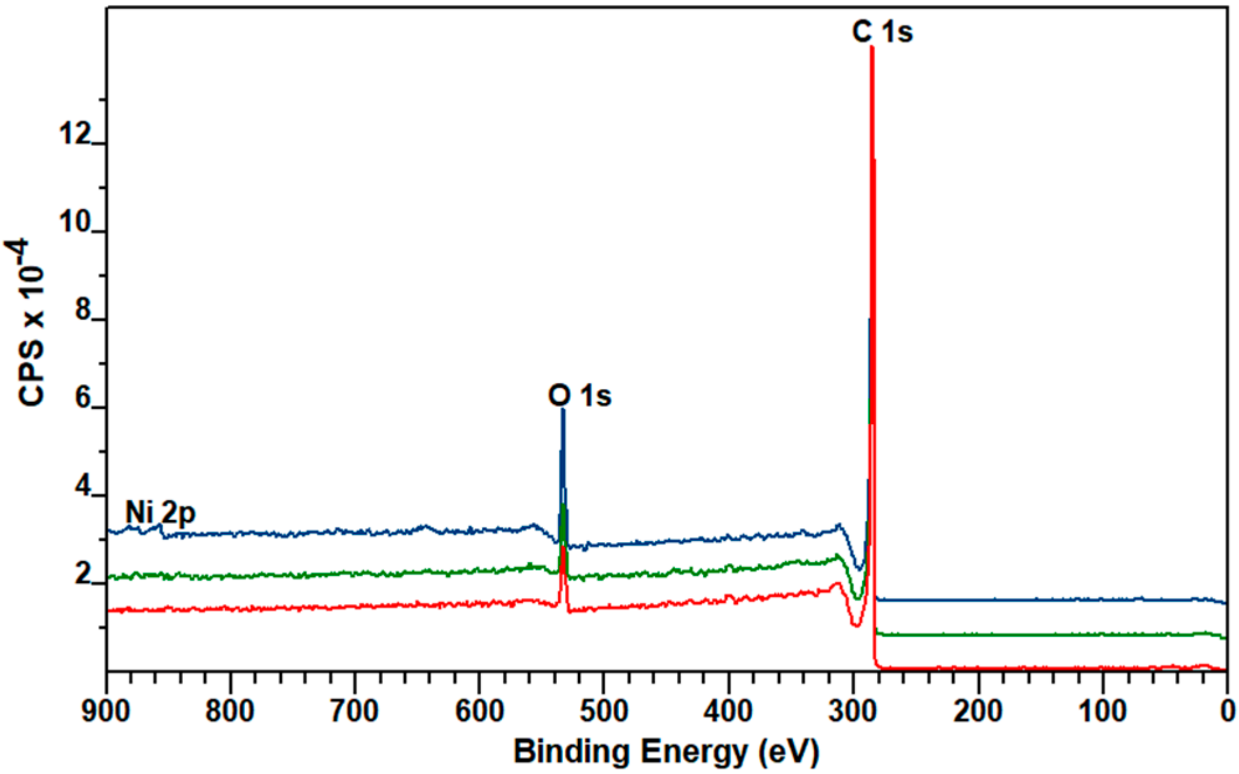

Figure S5: Survey XPS spectra of pristine HCSs (red), Ni@HCSs$_{Ni(acet)2}$ (blue), and Ni@HCSs$_{NiCl2}$ (green) nanocatalysts

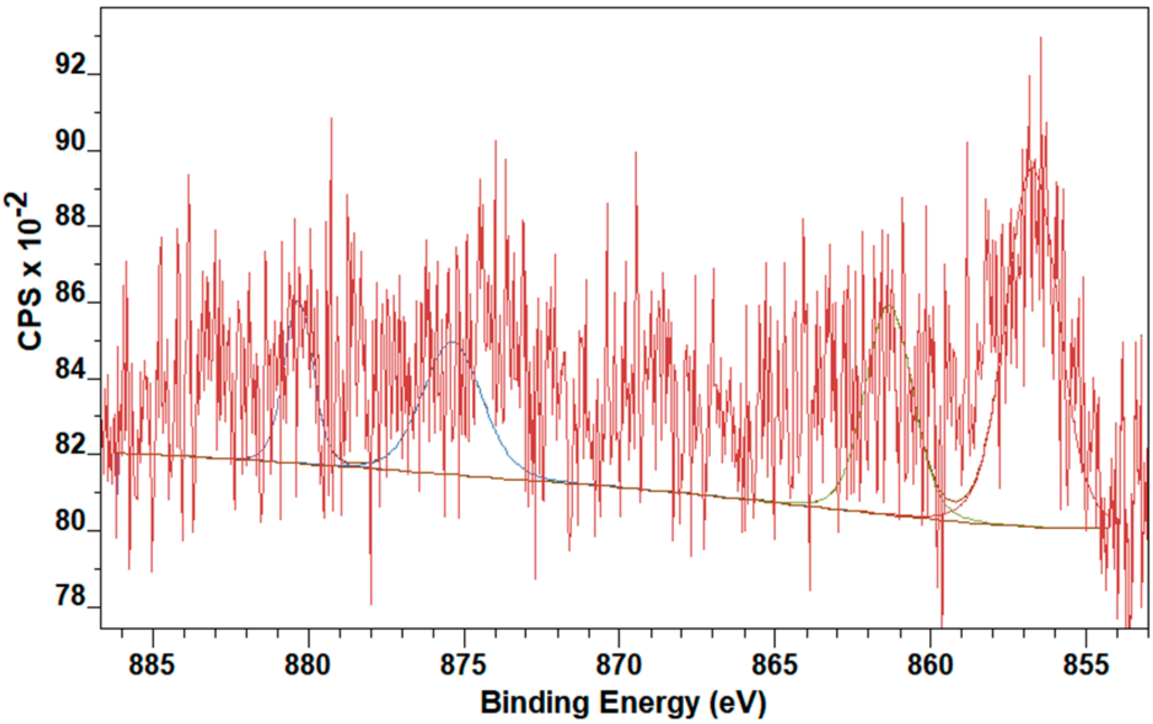

Figure S6: Deconvoluted Ni 2p spectrum of Ni@HCSs$_{NiCl2}$ nanocatalysts.

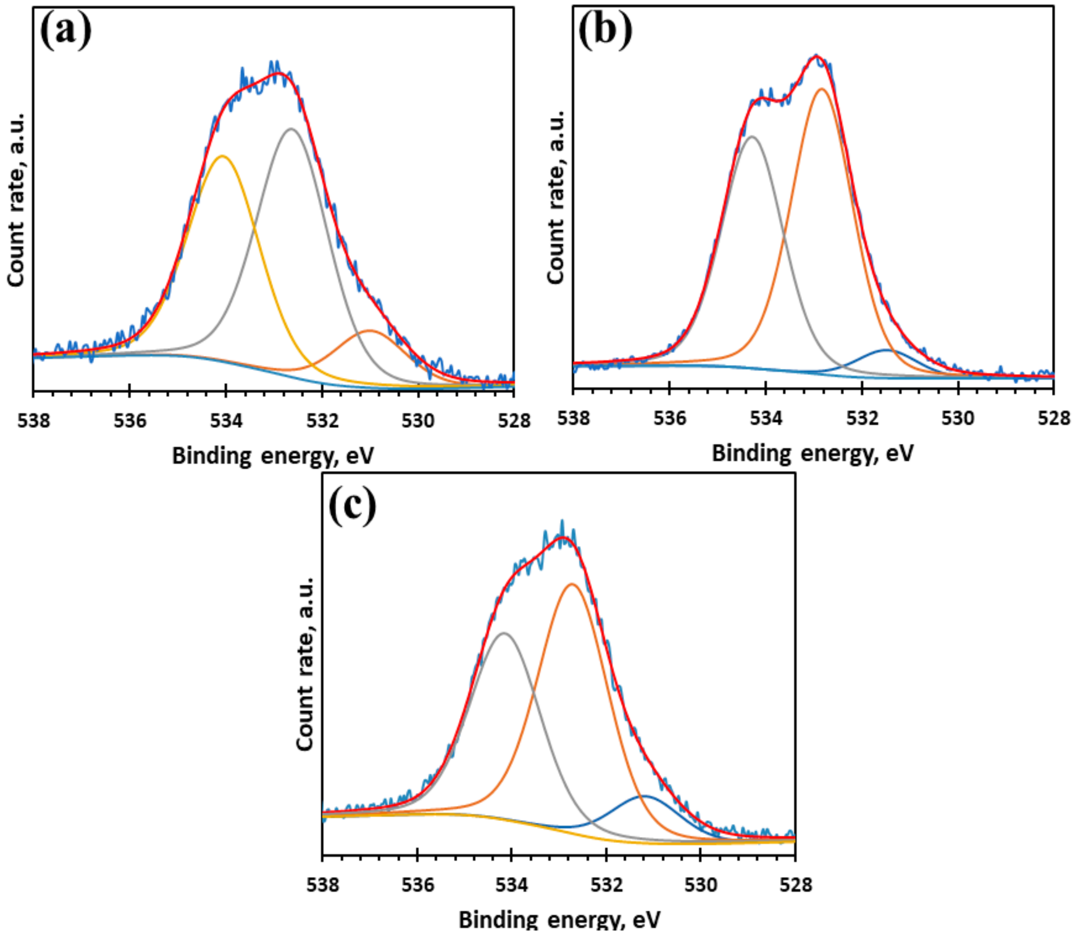

Figure S7: Deconvoluted O1s spectra of (a) pristine HCSs, (b) Ni@HCSs$_{Ni(acet)2}$, and (c) Ni@HCSs$_{NiCl2}$ nanocatalysts.

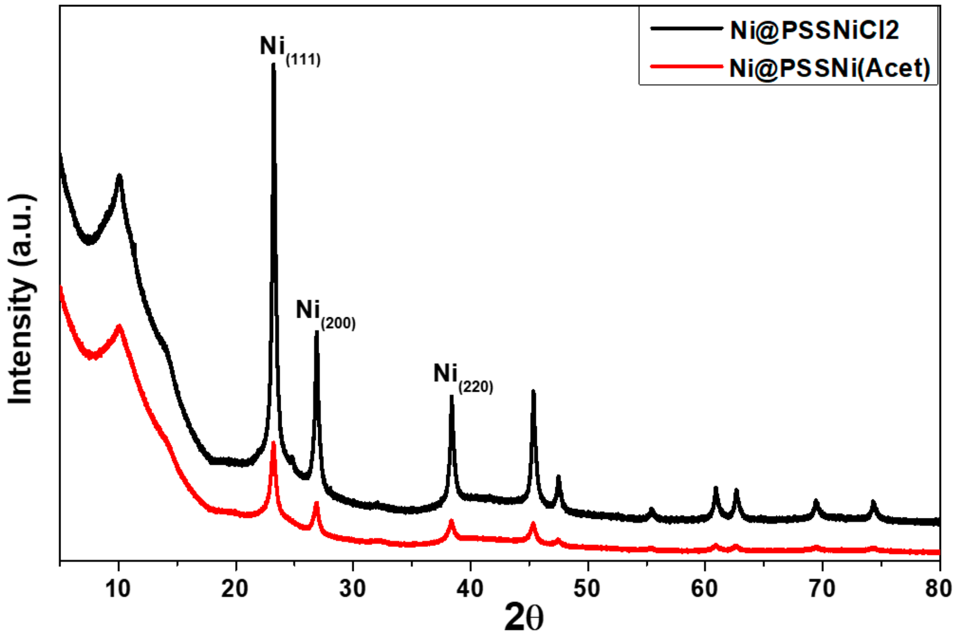

Figure S8: XRD patterns of the Ni nanoparticles on the PSS templates prior to RF coating.

Table S1: Morphological parameters based on TEM micrographs

Sample	Size (nm)				
	Diameter	Cavity	Shell	Ni nanoparticle	
				TEM	XRD
**HCSs**	342 ± 17	327 ± 16	10 ± 2.1	-	-
**Ni@HCSs$_{NiCl2}$**	332 ± 25	281 ± 21	23 ± 4.7	12 ± 1.9	10.9
**Ni@HCSs$_{Ni(acet)}$**	326 ± 25	245 ± 19	42 ± 6.9	6.4 ± 1.2	8.6

Table S2: Thermal stability properties of pristine and Ni@HCSs samples

Sample	*Onset Temp (°C)*	*Decomp. Temp. (°C)*			*% Residue*
		(i)	(ii)	(iii)	
HCSs	568.1		642.7		0.76
Ni@HCSs$_{Ni(acet)}$	452.2	489.3	582.6	630.5	6.84
Ni@HCSs$_{NiCl2}$	426.1	484.5	552.5	592.9	10.2
